# PHOTO QUIZ

**DOI:** 10.3201/eid1606.091937

**Published:** 2010-06

**Authors:** Myron G. Schultz

**Affiliations:** Centers for Disease Control and Prevention, Atlanta, Georgia, USA

**Keywords:** Carrión disease, Daniel Alcides Carrión, Oroya fever, verrugans peruana, Bartonella bacilliformia, bacteria, vector-borne infections, photo quiz

**Figure:**
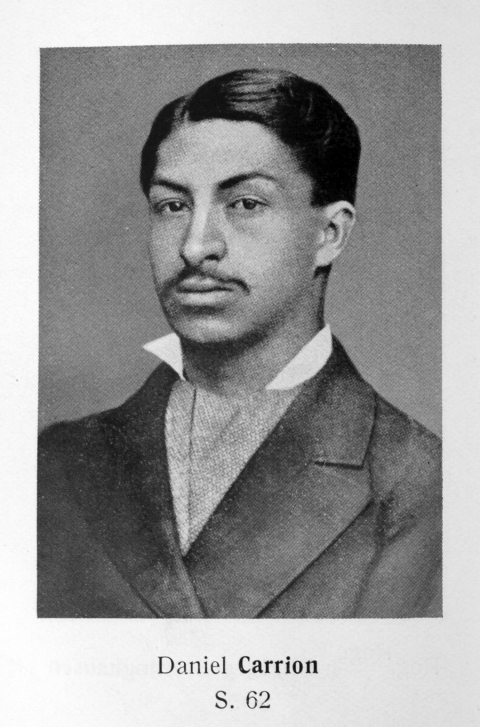
Who is this man?

Here is a clue: He died in a self-experiment that showed that verruga peruana and Oroya fever are etiologically related.

## Who is he?

Albert Barton
Daniel Alcides Carrión
Max Kuczynski-Godard
Ernest Odriozola
Garcilasco de la Vega
