# Supplementary material for: PHOTO QUIZ
Source: Emerg Infect Dis. 2010 Jun;16(6):1025–7. doi: 10.3201/eid1606.091937 (PMC3086236; doi:10.3201/eid1606.091937)
Supplement: Related texts

# Other: Daniel Alcides Carrión

**Published:** 2010-06

**Authors:** Myron G. Schultz

**Affiliations:** Centers for Disease Control and Prevention, Atlanta, Georgia, USA

**Figure:** This is a photograph of Daniel Alcides Carrión (1858–1885)

Carrión was a medical student in Lima Peru. He died from an experiment that he performed on himself when he was 26 years old. Carrión’s experiment showed that 2 strikingly different diseases endemic to Peru, verruga peruana and Oroya fever, are etiologically related to each other. In the light of modern knowledge, we now know that these 2 syndromes, 1 benign, the other pernicious, are both caused by the hemotrophic bacterium, *Bartonella bacilliformis*. Verruga peruana and Oroya fever are now called Carrión disease. Carrión’s motives for his self-experiment were initially misunderstood by his contemporaries, and subsequently by some medical historians. Nonetheless, his self-sacrifice has been honored by Peruvians for the past century, and he is the foremost hero of Peruvian medicine. The day of his death, October 5, is celebrated yearly as the “Dia de la Medicina Peruana,” and the Peruvian National University in Cerro de Pasco carries his name.

From pre-Inca times, through the Spanish Conquest, to the “Guano Age” of the 19th century and up to the modern period, Carrión disease has played a role in the life of Peru. It is distributed in the Andean cordillera in certain mountain valleys that are positioned at right angles to the prevailing wind. It is in this special environment that the diminutive sandfly vectors, of which *Lutzomyia verrucarum* (formerly *Phlebotomus verrucarum*) is the most important species, can survive.

Verruga peruana, which is usually a benign condition, is clinically manifested as multiple eruptions of disfiguring, hemangioma-like tumors. Oroya fever, in contrast, is a disease of fever and anemia, with a high case-fatality rate. The disease acquired its name from the epidemic in 1871 that struck the workers on the Oroya railroad line at Cocachacra, 40 miles from Lima and 5,000 feet above sea level. It was commonly said that “every tie in the railroad had cost a life.” Although this disease became known as Oroya fever, it occurred at a considerable distance from the city of Oroya and it has never been contracted there.

When Daniel Carrión undertook his experiment, he was in his sixth year of training at the Facultad de Medicina in Lima. To qualify for his medical degree he was required to prepare an original thesis, and he had devoted himself with increasing vigor during the preceding 3 years to a study of the epidemiology and clinical manifestations of verruga peruana. It was a work in which he invested his emotions as well as his intellect. As a young boy, he had made frequent trips with his uncle through the Peruvian mountains, going to and from school in Lima to his home in Cerro de Pasco, a mining town at the height of the Andes. He saw people with verrugas during these trips, which made a deep impression on him. He later told a classmate that through his research he hoped “to make an important contribution to aching humanity.”

As his studies progressed, Carrión became concerned with the difficulty in diagnosing verruga peruana before the eruption appeared. He was vaguely aware that the appearance of the verrugas was preceded by fever and anemia, but his chief concern was the confusion between the prodromal phase of verruga and other febrile disorders, particularly malaria. Carrión thought that if verruga peruana could be recognized early enough, more effective treatment could be applied by the local practitioners. Gradually, Carrión came to the conclusion that the most effective way to study the incubation period and symptoms of verruga peruana would be to inoculate himself. As the months went by, his determination to do this became more intense. He often spoke of his plan to his friends and professors, and they repeatedly tried to dissuade him. When the Academia Libre de Medicine set up a prize competition on the subject of verruga peruana, Carrión could no longer be stopped.

On the morning of August 27, 1885, Carrión was in the Nuestra Seńora de las Mercedes ward of the Dos de Mayo Hospital in Lima. In bed no. 5 was Carmen Paredes, a 14-year-old boy with a verruga on his right eyebrow. The boy was anxious to leave the hospital, and Carrión became concerned that he would miss the chance to inoculate himself before his forthcoming vacation period. Using a lancet, Carrion tried to inoculate his arm with blood taken from the verruga. He succeeded with the assistance of Dr Chavez, a young ward physician.

Although Carrión kept a diary of his clinical illness, he did not record his thoughts until the first symptoms appeared, 21 days after the inoculation. On September 17, Carrión felt discomfort and pain in his left ankle. Two days later, fever began. He also experienced chills, abdominal cramps, and pain in all the bones and joints in his body. He was unable to eat anything, and he commented in his diary that his thirst was devastating. His urine became dark red and scanty, and his skin took on an icteric tint. By September 26, he had become too weak to maintain his diary. This task was taken up by his classmates who were keeping vigil at his bedside. They were amazed at how quickly anemia had developed. At the same time, a systolic murmur developed and grew in intensity. On October 1, fasciculations appeared in his arm muscles. Carrión’s health was failing, but he was thinking clearly enough to say to his friends: “Up to today, I thought I was only in the invasive stage of the verruga as a consequence of my inoculation, that is, in the period of anemia that precedes the eruption. But now I am deeply convinced that I am suffering from the fever that killed our friend, Orihuela. Therefore, this is the evident proof that Oroya fever and the verruga have the same origin, as Dr. Alarco once said.”

This remarkable insight expressed the essence of Carrión’s experiment. He had not, as is often said, set out to prove the single cause of verruga peruana and Oroya fever. He had merely intended to study the onset of verruga peruana; yet, when a completely different disease developed, he was able, despite his grave state, to grasp the full meaning of his experiment.

On the evening of October 3, Carrión was completely delirious and rambled on about the different opinions that existed on the pathology of verruga peruana. On October 5, thirty-nine days after the inoculation, he fell into a coma. Most of what he uttered was incomprehensible, but his last words were heard clearly by one of his friends. He said “Enrique, c’est fini [it is finished in French]*.*” At 11:30 pm he died.

Scientific understanding of the disease that killed Daniel Carrión has grown substantially in the 125 years since his death. The etiologic agent, vector, laboratory manipulation of the organism, treatment, and prevention of Carrión disease have all been elucidated. Carrión disease is recognized as 2 different clinical manifestations of human bartonellosis due to *Bartonella bacilliformis* infection. In recent years, bartonellosis has also been recognized as a group of emerging zoonotic infectious diseases comprised of at least a dozen species of the genus *Bartonella*, which can cause cat-scratch fever, trench fever, bacillary angiomatosis, and peliosis hepatitis.

Advances in medical science come about through the collective work of hundreds of thousands of investigators. Only on rare occasions do investigators dare to experiment on themselves and seldom do these experiments end fatally. Daniel Carrión is a martyr whose courage should be remembered by current and future generations. In his short life as an unknown medical student, he was able to fulfill his wish to “make an important contribution to aching humanity.” Not only did he demonstrate the unitary etiology of verruga peruana and Oroya fever, he also revealed the phenomenon that diverse diseases can be produced by a single pathogen.

## References

[1] Altman LK. Who goes first? New York: Random House; 1987. p. 1–5.

[2] Chomel BB, Boulouis H-J, Maruyama S, Breitschwerdt EB. *Bartonella* spp. in pets and effect on human health. Emerg Infect Dis. 2006;12:389–94.1670477410.3201/eid1203.050931PMC3291446

[3] Leonard J. Daniel Carrión and Carrión’s disease. Bull Pan Am Health Organ. 1991;25:258–66.1742572
